# AZDAST the new horizon in antimicrobial synergism detection

**DOI:** 10.1016/j.mex.2016.01.002

**Published:** 2016-01-07

**Authors:** Navid Ziaei-Darounkalaei, Mehrdad Ameri, Taghi Zahraei-Salehi, Omid Ziaei-Darounkalaei, Tahereh Mohajer-Tabrizi, Lotfollah Bornaei

**Affiliations:** a Department of Veterinary Medicine, Faculty of Agriculture, Islamic Azad University, Yasuj Branch, Yasuj, Iran; b Department of Pathology, Amgen Inc., USA; c Department of Microbiology, Faculty of Veterinary Medicine, University of Tehran, Tehran, Iran; d Department of Orthopedic Surgery, Tehran University of Medical Sciences, Tehran, Iran; e Department of General Surgery, Gorgan University of Medical Sciences, Gorgan, Iran; f Department of Animal Science, Faculty of Agriculture, Islamic Azad University, Yasuj Branch, Yasuj, Iran

**Keywords:** AZDAST antibiotic double combination

## Abstract

The attempts via introducing many methods have been conducted to select the best antibiotic combination in the treatment of seriously ill patients. Operational or interpretational complexity or time-consuming along with sufficient accuracy led to postpone routine clinical use of these tests until today, despite the urgent need for them. By this study and proposed method, selection of the best double antibiotic synergistic combination against resistant pathogen is simply same as Kirby-Bauer antibiotic susceptibility test. It seems, precise and reliable results (very low coefficient of variation) will be introduced it as a routine accurate diagnostic doubled antimicrobial synergism test.

• The objective of this study was to introduce a novel method in antibiotic interaction detection.
• It demonstrates high sensitivity and accuracy.
• Easy implementation by routine microbiology labs materials and equipment and so easy stand-alone interpretation seems to make it friendly test be able to replacing the previous methods.

## Graphical abstract

**Figure:**
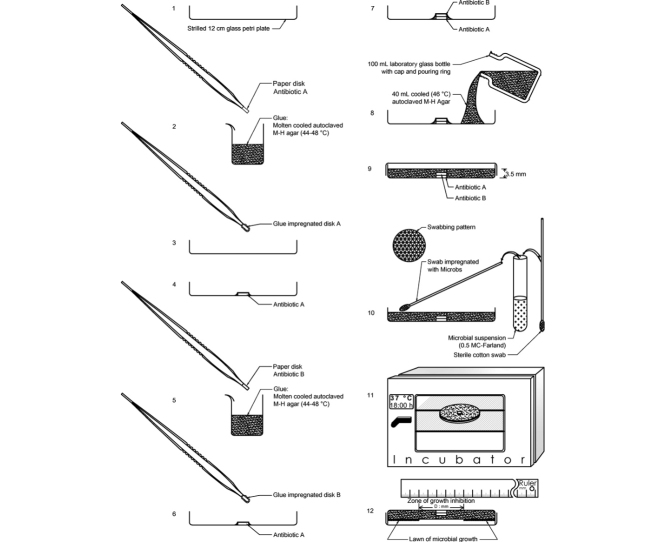

## Method details

This is a novel antimicrobial synergism evaluating method. Recent developments in clinical microbiology, emphasizes the justified role of laboratory on assaying antibiotic combinations [20]. This method could be categorized in the disk diffusion double antibiotic synergism tests. To distinguish from older DASTs, new method named Ameri-Ziaei double antibiotic synergism test (AZDAST).

The diameter of zones of growth inhibition is creating data like in other disk diffusion methods. Hence, everyone able to performing AZDAST as Kirby-Bauer disk susceptibility test. On the other hand, AZDAST is a standalone test that interpreted on the basic pharmacological definitions listed in Table 2.

### Equipment and materials requirements

A brief summary of what is needed for implementation of the new method was listed below. The listed requirements are available in every microbiology laboratory.

Equipment

**Table:** 

Spectrophotometer	(OD_625_) or 0.5 McFarland turbidity standard
Autoclave	
Magnetic Heater Stirrer	Adjusted on 44–48 °C approximately
Water bath	(44–48 °C)
Incubator	(Gravity convection microbiology incubator)
Graduated cylinder	(50 mL)
Autoclavable glass bottle	(100 mL with cap and pouring ring)
Glass petri plate	(12 cm)

Materials

**Table:** 

Antibiotics paper disks	(Padtan-TEB Co., Tehran, Iran)
Mueller-Hinton agar	(Merck KGaA, Darmstadt, Germany)
Samples	Patients pathogens (Clinical referrals)

### Method procedure and interpretation

The following steps should be carried out to run AZDAST, in the same order shown in graphical abstract.

**Fig. 1:**
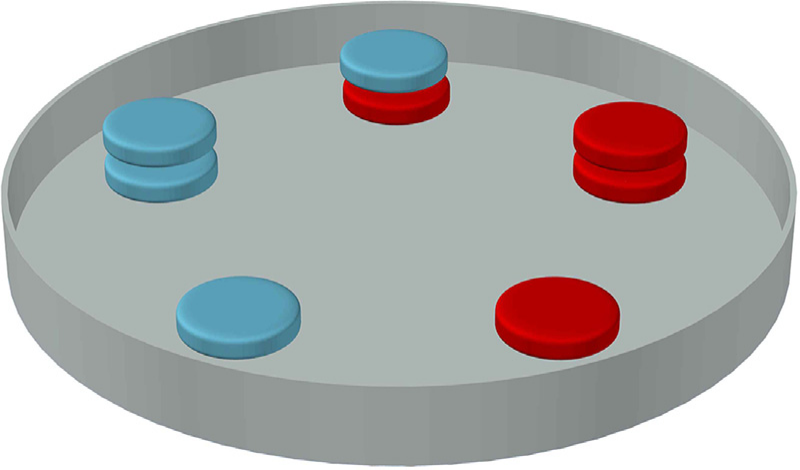
Completed AZDAST petri plate before pouring agar: Top-center: combination position, right and left doubled: cummulation positions and down right and left single: deep Kirby-Baure disk susceptibility test.

**Fig. 2:**
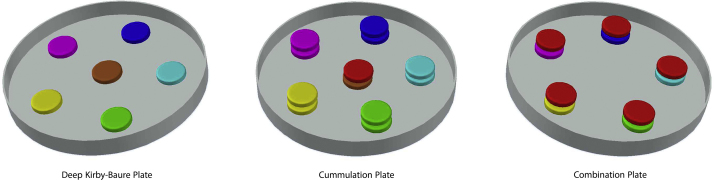
Compositional separate plates of AZDAST consist of deep Kirby-Baure plate, cummulation plate and combination plate.

1. Provide a sterile glass petri dish with the diameter of 12 cm (Graphical abstract: step 1).
  Notice: Another size and disposable petri dishes could be selected to perform AZDAST. Here, must comply regulations such as agar depth, etc.
2. Make an adhesive to past antibiotic paper disk on the floor of the petri dish. The glue contains the 1.5 times concentration molten cooled (44–48 °C) autoclaved Mueller-Hinton agar. Maybe put the cooled agar on the heater to avoiding solidification.
3. Dip the first antibiotic paper disk in the glue (i.e. “A” disk in the graphical abstract step 2).
4. Paste the smeary “A” disk in an intended place on the floor of the petri dish (Graphical abstract: steps 3 and 4).
  Notice: Only combination site of dish has been shown in the graphical abstract exemplar. All the positions shown in the following pattern must be pasted before agar pouring (so-called AZDAST petri dish) (shown in Fig. 1).
5. Similarly, the second disk (“B”) smeared and pasted in its predetermined places (Graphical abstract: steps 5, 6 and 7).
6. Now, the petri is filled by 40 mL of lukewarm (Graphical abstract: step 8) (heat-out via 46 °C shaking water bath for 30 min) autoclaved Mueller-Hinton culture medium. The agar depth should be 3.5 mm.
  Notice: Agar depth ranging three to four mm is acceptable (Details were not presented in this paper).
  Notice: Synergism detection among several antibiotics could be design in several plates or could be perform in the separate plates compositionally, as **combination plate**, **cummulation plate** and **deep Kirby-Baure plate** to easier to disk pasting arrangement. However, equal agar depth must be prepared in compositional separate plates, as described in Fig. 2.
7. Let the agar solidified within a few minutes. Now, the *AZDAST petri plate* is ready to inoculate (Graphical abstract: step 9).
8. The inoculum prepared from direct colony suspensions of a fresh 24-h culture, equivalent to a 0.5 McFarland standard. Equal optical density of 0.08–0.10 at wavelength of 625 nm performed in this study.
9. The plate inoculated using spread plate technique by a sterile swab (Graphical abstract: step 10).
10. Incubate the plate at 37 °C for 18 h (Graphical abstract: step 11).
  Notice: The rule of 15, 15, and 15 could and should be followed [24].
11. Similarly to the Kirby-Bauer method (CLSI guideline), the diagonal of the zone of inhibition could be measured by a ruler or caliper (Graphical abstract: step 12).
12. The obtained values of diameter of zones of growth inhibition are interpreted based on Table 1.

Notice: The AZDAST is standalone without a series of reference tables. Interpretation is based comparing the diameters of zones of single and dual disks (doubled dose, and combined). This syllogism produced the final interpretation according to Table 1.

Notice: The synergism has not been an individual's preference, agenda, or wishes. The combination index ranges from <1, =1, and >1 indicate synergism, additive effect, and antagonism, respectively. By this log (CI) grading, synergism is subdivided into (near) additive (±), slight synergism (+), moderate synergism (+ +), synergism (+ + +), strong synergism (+ + + +), and very strong synergism (+ + + + +). Antagonism is divided in the same way, except using “−” sign(s); thus, the corresponding symbols are ±, −, − −, − − −, − − − −, and − − − − − [4].

**Fig. 3:**
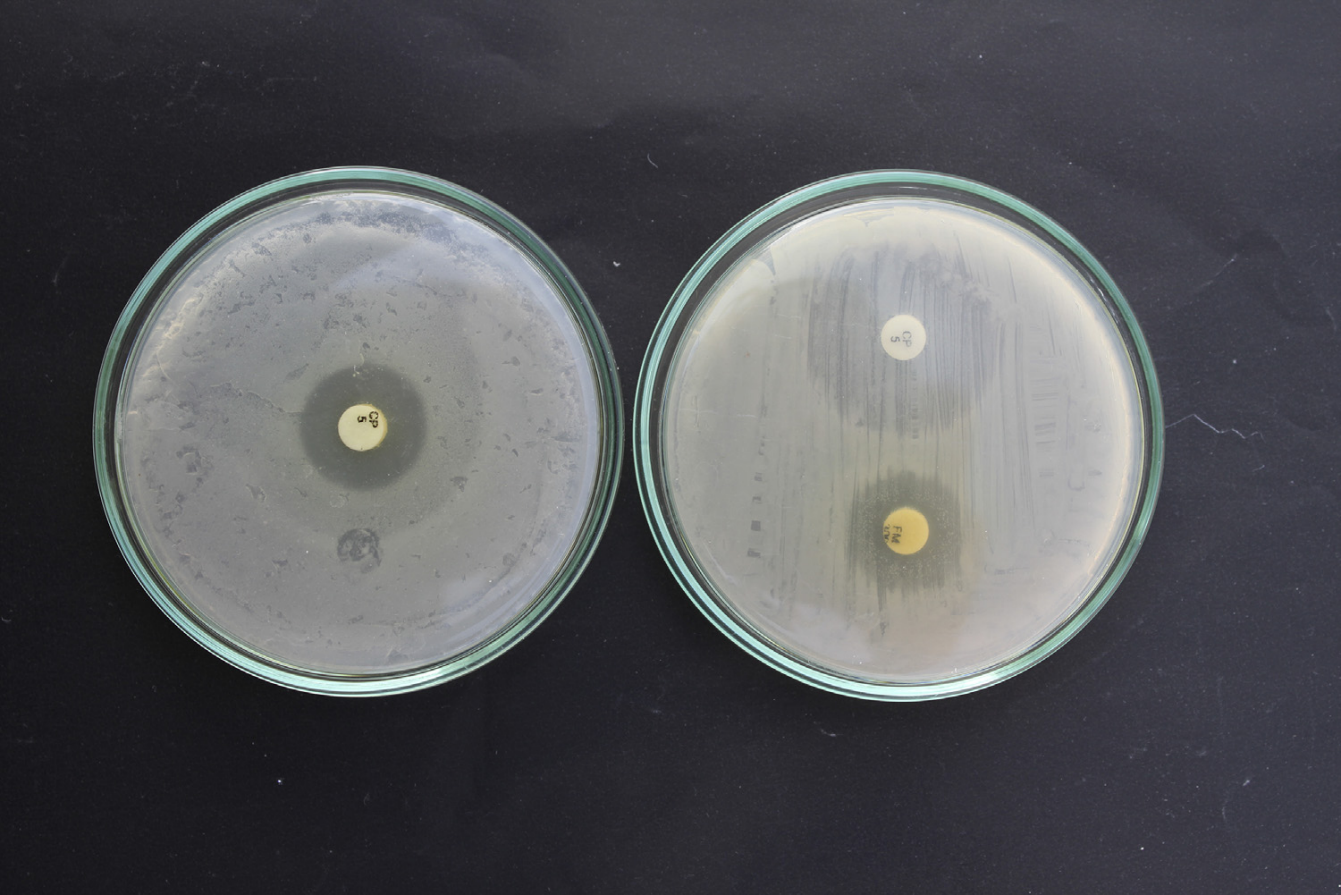
An impressive synergism has been shown against a multiresistant wild *E. coli* isolate by AZDAST. [This photo is the first try to implementing the idea of AZDAST.] Photo by authors.

Hereunder shown the first try to implementing the idea of AZDAST in the two separate 9 cm glass petri dishes. An impressive synergism has been shown between two resistant antibiotic against an *E. coli* isolated from a urinary infection patient (Fig. 3).

## Method validation, additional information and supplementary material

The factors may be affecting the new method has been studied. The new method ability on synergism/antagonism detection evaluated using two well-known antibiotic combinations (see Table 1). The disk approximation technique has validated the results of AZDAST on the combinations as golden method.

### Internal factors affecting new method

Many factors potentially could influence the results of a disk diffusion test. However, since the new method is performed as a stand-alone test with a unique and different procedure by performing in a day and a petri plate), main factors could not affect the results.

**Fig. 4:**
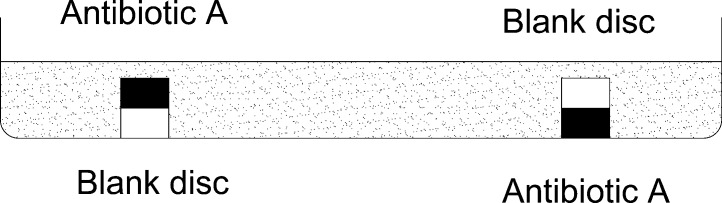
Pattern used for evaluating diffusion properties of antibiotics in the new method.

In this regard, *diffusion properties* such as size and charge of a drug molecule (two crucial properties involved diffusion) [24] are the similar situation in all cases of diffusion methods. On the other side, it may seem to affect the results by more distance of deeper disk from agar surface (lawn of bacterial growth) and blocking of its diffusion by upper one. These possibilities have been studied by using a blank and different antibiotic disks and bacteria using the following pattern (Fig. 4). In this study found no evidence of any significant difference between two locations (data have not shown here).

The extent of the inhibition zone affects by *disk size*. The wider disks produce larger zones. It is mandatory using of the same size paper disks for all location in the plate. *The nature of the paper* used in the preparation of the disk will influence the diffusion, too [24]. Although the relevant standards are followed for the preparation of disk, to achieve a reasonable result, it is better to using all the disks from a company (see Table 2, for the disks used in this study).

The intensity of the antibiotics interaction and interactivation could be influenced by themselves concentration [25]. The different amount of drug (so called potency, mass and strength) lead to changes in inhibition zone [24]. Similar to another disk diffusion methods, using similar brand disks and equal concentration of an antibiotic in plate could be solved the problem.

The size of inhibition zone proportion with the *agar depth* reversely. In less than 3 mm of depth, zone may be deferred plate to plate [9], [24]. The effects of agar depth from two to five millimeters have been studied and the detailed data has not shown here. Based on our study, although the AZDAST is a stand-alone test, it could be run on three to four millimeters of depth that not only affected by factors in the low agar depths and not much medium consumed.

The *critical time*, interval between the agar inoculation to incubation, is one of the most important criteria in Kirby-Bauer method [9] that affect the zone significantly [24]. By the AZDAST *critical concentration*, the concentration of antibiotics in agar necessary prior to beginning the bacterial growth [24] was achieved, since all the disks seeded on the floor of the petri dish prior to agar pouring, solidifying and inoculation. Agar has been solidified during less than four minutes, conforming the rule of 15 min (data has not shown here).

The definite role of *critical population* (or in other words the inoculant density) on the results of antibiotic susceptibility is evident [2] and likely be greater than other factors on the diameter of zone [24]. Performing AZDAST within a petri dish and preparing interpretation using the results obtained from this plate, ensures that these factors unable to affect AZDAST.

Although the antibacterial effects of beta-lactamase antibiotics, clindamycin and macrolides has considered time-dependent (*incubation time*) [10] and affected by *incubator temperature* [9], [24], incubation a few hours more than usual recommendations as well as changes in incubator temperature will not usually significantly influence the interpretation [7]. In the new method, AZDAST Petri is incubated entirely, therefore, all the interpretation criteria be affected during incubation alterations, and hence, changes regarding the final interpretation may not be seen.

*Zone edge* [24] and *wedge shape of agar* [1], [9] in a dish has same situation in all disk diffusion method. Rough edges of zones and wedge shape solidified medium make them difficult to interpret.

The traditional disk approximation technique required evaluating the zones of single disks in first day and subsequent approximation applying between them for synergism detection [5], [19], [24]. With this calculation, the old method was required two working days compared AZDAST, in addition the *distance between disks* has not calculated in AZDAST.

The results of all antibiotic susceptibility tests were affected by the medium. The *medium related factors* such as Ca^2+^, Mg^2+^, Zn^2+^, antagonists of folate synthesis inhibitors, thymidine, thymine, sodium chloride, pH, and additives defined growth supplement [23], [25] unable to affect interpretation of the AZDAT petri plate because of all comparing parameters located in a petri which in it these conditions is the same for deep Kirby-Baure, cummulation and combination disks.

### Equipment and materials used in the study

The materials used in this study are provided in Table 2. The required equipment was similar to those listed above.

As the benefits of the new method implies, it has a structural similarity to the routine single disk diffusion antibiogram. Hence, all the raw materials used in the AZDAST are similar to them, as listed in Table 2.

Present method displayed a low level of coefficients of variation in eight replicates (Table 3, Table 4). The diameter of zones of inhibition used for interpretation of combinations bolded in the mentioned tables.

On sensitive ATCC standard bacteria, AZDAST showed its competency to detecting synergistic and antagonistic combinations of penicillin G and gentamicin and erythromycin and clindamycin on *Staphylococcus aureus* and *Escherichia coli*.

### Reference test

To compare and validate outcomes of the new method, both bacteria and antibiotic combinations entitled in Table 2 have been run on the disk approximation [18] method as the reference test. In the following tables, *on mean* interpretation of the results has been derived from Table 3, Table 4.

According to Table 5, the old method has been unable to demonstrating synergistic combination of penicillin G and gentamicin [3], [11], [12], [13], [14], [16], [21] against *Staphylococcus aureus* and only showed this effect in half of the cases against *Escherichia coli* (Table 6).

In addition, while the new method has been demonstrated antagonistic combination of erythromycin and clindamycin in most cases, elder method said there is no antagonism and more threatening synergistic and additive effect for this well-known combination [6], [8], [15], [17], [22].

In the present study, a new method was introduced as an alternative to the disk approximation method for testing antimicrobial combination. This test has been provided the reliable results with high sensitivity and accuracy and easy to interpret, shown free of disorders caused by physicochemical and environmental factors and substances. More studies are required to compare this method with other combination sensitivity methods.

Advantages of the new method

1. The AZDAST is a numeral scaled qualitative method that interpreted on comparing the diameters of the zones of inhibition in millimeter.
2. This method could be performed prior to the time consuming quantitative methods. By the AZDAST, the strongest synergistic combination maybe selected primarily and then quantitative amount of each antibiotic could be calculated by quantitative methods.
3. AZDAST is a stand-alone interpreting test with no need to standard tables.
4. The new method was performed using routine available laboratory materials.
5. Everyone understanding the test procedure and interpretation. Therefore, its use is not limited to a reference or research laboratory.

## Figures and Tables

**Table 1:** Interpretation guideline of the new method.

The combination result from AZDAST petri plate	AZDAST interpretation on the combination	Equivalent definitions in slang	Examples	
			Antibiotics	Results (mm)
If the **AB** is greater than **A** & **B** and smaller or greater than **AA** and/or **BB**	Synergistic	1 + 1 = 3	Pen	**45**
			Gen	30
			**Pen** + **Gen**	**46**
			Pen + Pen	50
			Gen + Gen	33

If one of the **A** or **B** is equal to zero and **AB** is greater than **A** & **B** and smaller or greater than **AA** and/or **BB**	Potentiation (enhancement)	0 + 1 = 2	Pen	0
			Gen	**25**
			**Pen** + **Gen**	**26**
			Pen + Pen	0
			Gen + Gen	27

If the **AB** is smaller than **A** or **B** (or only smaller than greater one)	Antagonistic	1 + 1 = 0	Ery	32
			Clin	**40**
			**Ery** + **Clin**	**39**
			Ery + Ery	34
			Clin + Clin	43

If the **AB** is equal to **AA** and/or **BB** (Which one is greater than **A** and **B**)	Additive	1 + 1 = 2	Gen	25
			Tet	25
			**Gen** + **Tet**	**27**
			Gen + Gen	28
			**Tet** + **Tet**	**27**

If the **AB** is equal to one of the **A** or **B** (equal to the greater one)	Not distinguishable	1 + 0 = 1	Amp	10
			**Tet**	**25**
			**Amp** + **Tet**	**25**
			Amp + Amp	15
			Tet + Tet	27

If consider two drugs, as **a** and **b** with the zones of growth inhibition as **A** and **B** respectively along with the **AB** for the combination position and **AA** and **BB** for cummulation positions of **a** and **b** in the AZDAST petri plate. **Pen**, Penicillin; **Gen**, Gentamycin; **Ery**, Erythromycin; **Clin**, Clindamycin; **Tet**, Tetracycline; **Amp**, Ampicillin.

**Table 2:** Materials and details used in the present study (new method and disk approximation technique). All the listed materials are similar to the routine clinical disk diffusion antibiogram; only the ATCC bacteria have been used as inocula.

Parameter	Description	Manufacturer/specifications
Inoculums		
Bacteria	*Staphylococcus aureus*	ATCC 25923
	*Escherichia coli*	ATCC 25922
Colony suspension method	Direct/by saline suspension	0.9% NaCl (w/v)
Inoculum density	0.08–0.10 equiv. to the 0.5 McFarland	Spectrophotometer: PG Instrument, T80^+^, OD_625nm_
Antibiotics		
Antibiotics (paper disks)	Penicillin G (1 Unit) Gentamicin (10 μg) Erythromycin (5 μg) Clindamycin (2 μg)	Padtan-TEB Co., Tehran, Iran
Paper disk diameter	6.4 mm	
Paper disk thickness	1 mm	
Antibiotic combinations	Penicillin G and Gentamicin	Synergistic combination
	Erythromycin and Clindamycin	Antagonistic combination
Culture plates		
Petri plate diameter	12 cm/glass	TGI™
Petri plate material	Glass	
Medium	Mueller-Hinton agar	Merck KGaA, Darmstadt, Germany
Medium contributor	50 mL graduated cylinder	DURAN^®^ Measuring Cylinder
Autoclaving medium container	Autoclavable100 mL laboratory glass bottle with cap and pouring ring	TGI™
Volume of medium	40 mL	
Depth of medium	3.5 mm	Three to 4 mm is acceptable (data has not shown here), but in this study the approximate depth of 3.5 mm has been applied

**Table 3:** Results of new method on penicillin G and gentamicin on *Staphylococcus aureus* and *Escherichia coli*.

Bacteria	Analysis	Disk combinations in the AZDAST petri plate (mm)				
		Pen	Gen	Pen + Gen	Pen + Pen	Gen + Gen
*Staphylococcus aureus*	Means of zones of inhibition	**45.75**	30.38	**46.75**	50.00	33.00
	Standard deviation	1.83	2.07	2.05	2.67	2.27
	Coefficient variation	0.04	0.07	0.04	0.05	0.07

*Escherichia coli*	Means of zones of inhibition	0.00	**25.88**	**26.63**	0.00	27.75
	Standard deviation	0.00	0.64	0.74	0.00	0.71
	Coefficient variation	0.00	0.02	0.03	0.00	0.03

**Pen** is a symbol of first antibiotic disk (Penicillin G, 1 Unit) and **Gen** for second one (Gentamicin, 10 μg). Petri dish 12 cm, medium volume: 40 mL, medium depth: 3.5 mm.

**Table 4:** Results of new method on erythromycin and clindamycin on *Staphylococcus aureus* and *Escherichia coli*.

Bacteria	Analysis	Disk combinations in the AZDAST petri plate (mm)				
		Ery	Clin	Ery + Clin	Ery + Ery	Clin + Clin
*Staphylococcus aureus*	Means of zones of inhibition	32.13	**40.38**	**39.00**	34.50	43.88
	Standard deviation	0.83	1.41	1.93	1.93	1.81
	Coefficient variation	0.03	0.03	0.05	0.06	0.04

*Escherichia coli*	Means of zones of inhibition	**9.50**	0.00	**8.13**	12.25	0.00
	Standard deviation	1.07	0.00	3.36	1.28	0.00
	Coefficient variation	0.11	0.00	0.41	0.10	0.00

**Ery** is a symbol of first antibiotic disk (Erythromycin, 5 μg) and **Clin** for second one (Clindamycin, 2 μg). Petri dish: 12 cm, medium volume: 40 mL, medium depth: 3.5 mm.

**Table 5:** Comparison table of two methods on detecting synergistic combination of penicillin G and gentamicin.

Bacteria	Cases/incidence rate	AZDAST				Disk approximation			
		Syn	Ant	Add	ND	Syn	Ant	Add	ND
*Staphylococcus aureus*	Total (No)^a^	6	2	0	0	0	0	8	–
	Incidence (%)	75	25	0	0	0	0	100	–
Overall interpretation	On incidence	Syn				Add			
	On mean^b^	Syn				–			

*Escherichia coli*	Total (No)^a^	3	0	0	5	4	0	4	–
	Incidence (%)	38	0	0	63	50	0	50	–
Overall interpretation	On incidence	Syn				Syn and/or ND			
	On mean^b^	Syn				–			

Syn: synergistic, Ant: antagonistic, Add: additive, ND: not distinguishable.

a 8 plates in each group have been inoculated.

b DATA were dedicated from Table 3.

**Table 6:** Comparison table of two methods on detecting antagonistic combination of erythromycin and clindamycin.

Bacteria	Cases/incidence rate	AZDAST				Disk approximation			
		Syn	Ant	Add	ND	Syn	Ant	Add	ND
*Staphylococcus aureus*	Total (No)^a^	3	5	0	0	1	0	7	–
	Incidence (%)	37.5	62.5	0	0	13	0	88	–
Overall interpretation	On incidence	Ant				Add			
	On mean^b^	Ant							

*Escherichia coli*	Total (No)^a^	2	4	0	2	0	0	8	–
	Incidence (%)	25	50	0	25	0	0	100	–
Overall interpretation	On incidence	Ant				Add			
	On mean^b^	Ant							

Syn: synergistic, Ant: antagonistic, Add: additive, ND: not distinguishable.

a 8 plates in each group have been inoculated.

b DATA were dedicated from Table 4.
